# Synaptic protein expression remodeling in the LOAD2 mouse model

**DOI:** 10.1002/alz70855_102380

**Published:** 2025-12-23

**Authors:** Claudia Rangel‐Barajas, Abigail Perkins, Dalia Elkhatib, Christopher D Lloyd, Cinthia Ingraham, Gareth R Howell, Michael Sasner, Gregory W Carter, Bruce T. Lamb, Adrian L Oblak

**Affiliations:** ^1^ Stark Neurosciences Research Institute, Indiana University School of Medicine, Indianapolis, IN, USA; ^2^ Indiana School of Medicine, Indianapolis, IN, USA; ^3^ The Jackson Laboratory, Bar Harbor, ME, USA; ^4^ Indiana University School of Medicine, Indianapolis, IN, USA

## Abstract

**Background:**

Alzheimer's disease (AD) is a neurodegenerative disorder characterized by progressive cognitive decline. Synaptic degeneration, a hallmark of AD in both humans and preclinical models, is considered one of the most predictive pathological features of cognitive decline and may even precede the formation of amyloid plaques. In this study, we aimed to investigate whether synaptic integrity and transcriptomic signatures related to synaptic function are disrupted in the brains of LOAD2 mice—a triple homozygous model developed by the MODEL‐AD Center, carrying the APOE4, Trem2*R47H, and hAβ genetic risk variants.

**Methods:**

Subcellular fractions were isolated from LOAD2 mouse brains to separate synaptic PSD95‐enriched fractions and extra‐synaptic fractions. The expression of proteins involved in synaptic structure and function was evaluated at four different ages using Western blot analysis. To explore gene expression changes associated with neuropathology, Nanostring multiplex nucleic acid hybridization technology was employed for comprehensive gene profiling.

**Results:**

LOAD2 mice displayed significant changes in the expression of presynaptic and postsynaptic proteins. While synaptophysin, Homer1, and PSD95 were not altered in either 12‐ or 18‐month‐old LOAD2 mice, significant age‐dependent changes were observed in the presynaptic proteins SV2A and bassoon. Importantly, we also identified substantial changes in the subunit composition of NMDA and AMPA receptors at postsynaptic sites in LOAD2 mice. Additionally, expression levels of neurogranin, a critical synaptic protein marker that is decreased in the cerebrospinal fluid (CSF) of AD patients, were notably decreased in LOAD2 mice.

**Conclusions:**

The MODEL‐AD Center has developed mouse models with humanized genetic risk factors to better study the progression of late‐onset Alzheimer's disease (LOAD). Our findings demonstrate that the synaptic protein alterations in the LOAD2 mouse model, closely resemble the synaptic signatures observed in AD pathology.

